# A Case Report of Brucellosis-Associated Infective Endocarditis

**DOI:** 10.7759/cureus.37407

**Published:** 2023-04-10

**Authors:** Syed Muhammad Hussain Zaidi, Peter A Iskander, Muhammad Saad Choudhry, Anthony Iskander, Khalid Ahmed, Simin Nasar, Douglas Klamp

**Affiliations:** 1 Internal Medicine, Dow University of Health Sciences, Civil Hospital Karachi, Karachi, PAK; 2 Internal Medicine, The Wright Center for Graduate Medical Education, Scranton, USA; 3 General Surgery, Dow University of Health Sciences, Civil Hospital Karachi, Karachi, PAK; 4 Internal Medicine, Xavier University School of Medicine, Oranjestad, ABW; 5 Family Medicine, The Wright Center for Graduate Medical Education, Scranton, USA

**Keywords:** unpasteurized dairy products, veterinarians, valvular vegetation, endocarditis, brucellosis

## Abstract

Brucellosis is a prevalent zoonotic infection that can be relatively well managed and tolerated if appropriate treatment is initiated. Unfortunately, likely secondary to decreased awareness and vague symptoms, the diagnosis can be easily missed leading to worsening complications that severely increase the mortality rate. We present a case of a 25-year-old female who presented from a rural setting with a diagnosis of brucellosis, which was delayed. She ultimately developed infective endocarditis with cardiac vegetations on imaging. Despite improvement on antibiotics and reduction in size of cardiac vegetation, she suffered a fatal cardiac arrest before undergoing surgical intervention. Better awareness regarding hygiene and sanitary food handling should be encouraged, especially in underdeveloped rural areas, to help prevent infection. More studies need to be performed to help better identify symptoms coupled with maintaining a high index of suspicion so as to expedite diagnosis, treatment, management and hopefully prevent the progression of disease and worsening complications.

## Introduction

Brucellosis is a notorious worldwide zoonotic infection that can be difficult to diagnose. It is one of the most common zoonotic diseases in the world, with an incidence of more than 500,000 cases annually. The etiology of the illness is predominantly zoological in nature [1]. This means that transmission can be due to direct contact (veterinarians, livestock keepers) as well as consumption of unpasteurized dairy products. Brucellosis has a profound inclination towards developing nations, and although the untreated case-fatality ratio hovers at 1-5%, the extended disease course and complications necessitates prolonged therapeutic intervention. The presentation of the illness is usually vague with non-specific symptoms including fever, headache, malaise, loss of appetite, which often delays diagnosis. Infective endocarditis (IE) is one of the several complications associated with this condition and counts towards 80% of fatalities associated with this disease overall [2]. The incidence of brucellosis infection in Pakistan is substantially underestimated, and neighboring countries like India have also documented numerous cases; however, no authoritative statistics have been reported [1]. In areas where lack of awareness exists, the diagnosis and therefore treatment process becomes hindered and delayed. Another risk factor for brucellosis in developing countries is absence of hygienic measures during childbirth. Since the majority of patients recover spontaneously, with only 4% developing severe complications like infective endocarditis, the infection is not always taken seriously despite a mortality rate of up to 80% when complications occur [2]. We report a case of brucellosis infection in a young woman who came to the ER of Civil Hospital Karachi (CHK) with symptoms of fever, altered level of consciousness, weight loss, and fatigue after delivering a baby vaginally two and a half months prior. She was diagnosed with Brucella-associated infective endocarditis after an enzyme-linked immunoassay (ELISA) test for Brucella antibody came back positive. Despite appropriate treatment she unfortunately suffered a fatal cardiac arrest within one week.

## Case presentation

**Figure 1:**
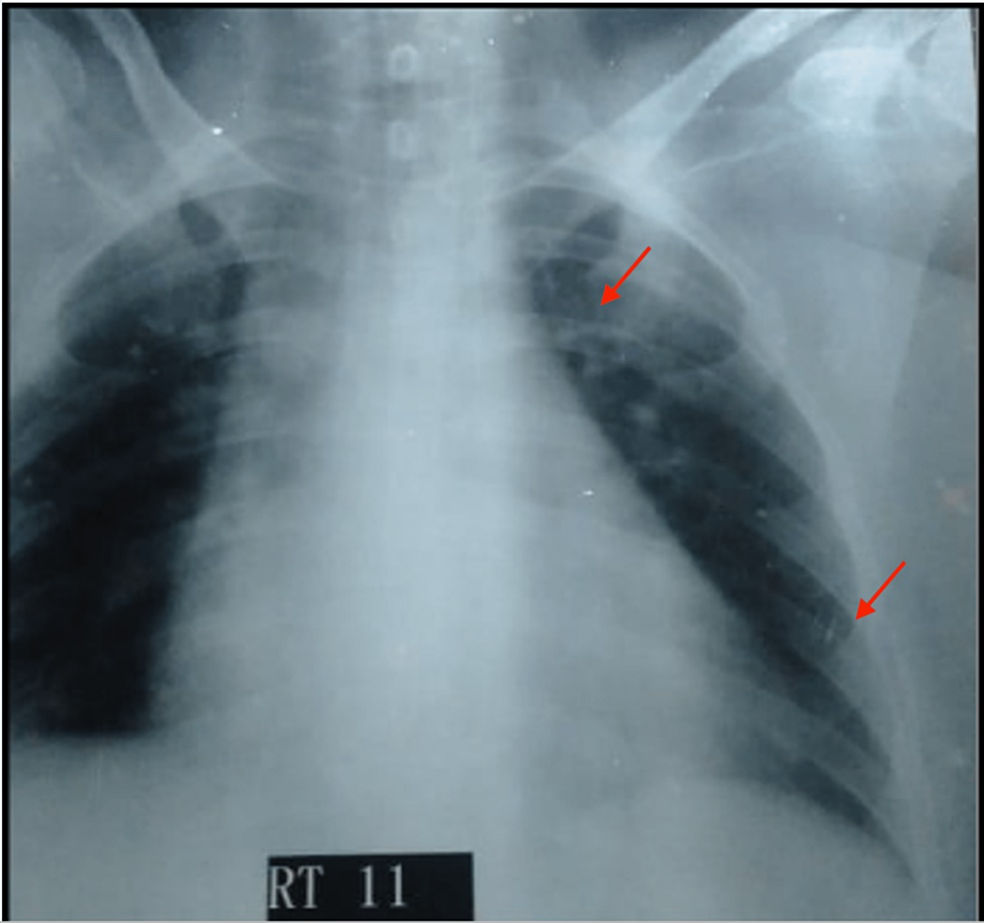
Cardiomegaly and a calcified foci in the left upper zone

A 25-year-old female resident of Larkana, who worked in animal farms, presented in the emergency department with a two-month history of fever, along with right-sided weakness and altered level of consciousness for one week. The patient had a normal vaginal delivery roughly 2.5 months before, followed by the onset of sudden rigors, chills, and documented fevers up to 103°F. Fever was intermittent and was relieved with antipyretics. The patient also reported a decrease in appetite, weight loss, body pain, headache, and fatigue for the last two months. She subsequently developed right-sided weakness. Gradually, her level of consciousness began to alter, progressing to a state of disorientation without evidence of dizziness, vertigo, double vision, seizures, or vomiting. The patient had a normal gynecological history and had delivered two daughters through spontaneous vaginal delivery in the past. Her vitals included blood pressure 141/84 mmHg, pulse 100/min, respiratory rate 21/min, and temperature 100.1 F. Physical exam was significant for palpebral conjunctival pallor, decreased tone and reflex response in extremities. Heart examination revealed normal sounds with no murmurs. Laboratory values on admission were significant for white blood count (WBC) 18.4*10E9/L, hemoglobin 6.5g/dl, hematocrit 22.4%, mean corpuscular volume (MCV) 64.9 fL, erythrocyte sedimentation rate (ESR) 46mm/hr, and C-reactive peptide (CRP) 225.4 mg/dl. She had an iron panel indicating anemia of chronic disease. Thrombophilia profile and malaria screen were negative. Chest X-ray was concerning for cardiomegaly and left upper lobe calcified foci (Figure 1). Magnetic resonance imaging of the brain showed multiple small infarcts and an old infarction in the right temporo-parietal region. Attenuation of the M3 segment of the left middle cerebral artery on MRI was noted, likely due to stenosis. 

An echocardiogram of the heart was done which showed a thickened mitral valve with mild grade 2 regurgitation more than stenosis. A mobile echogenic mass was noted on the valve measuring 12.76 mm by 11.86 mm. As per infectious endocarditis protocol, three sets of blood cultures were sent from different sites drawn one hour apart. She was started on intravenous (IV) vancomycin 1g every 12 hours (Q12H), IV ceftriaxone 2g once a day, and IV gentamycin 60 mg every eight hours (Q8H). Due to negative blood cultures and history indicating residence in a rural area with livestock. The brucellosis antibody titer was 55 IU/ml of ELISA-IgG. She was switched to oral doxycycline 100mg, oral rifampicin 600 mg, and IV ciprofloxacin 400 mg Q12H. Repeat echocardiogram one week later showed improvement of the mass to 5.9 mm by 8 mm. The patient was scheduled for surgery, however, she unfortunately experienced an irreversible cardiac arrest.

## Discussion

The prevalence of brucellosis infection in developed countries is low [2]. The highest prevalence is reported in the Mediterranean region, whereas the subcontinent region of Pakistan and India, although being endemic is unreported due to scarcity of data although cases in literature are found [1]. Initial reports like Tamimi Raju et al. said that the exact prevalence of the disease in India is unknown, likely secondary to population size and decreased awareness. However a seroprevalence study by Senthil et al. on bovine brucellosis in Chennai, India utilizing various diagnostic tests with positive results ranging from 3.3%-11.4% [3]. This may be one reason why some areas, such as Pakistan, tend to miss the diagnosis of brucellosis. Higher numbers have been reported, however, in areas such as the Arabian Peninsula, the Mediterranean, Mexico, as well as Central/South America [4]. Due to non-specific symptoms, such as fever and lethargy, there is often a delay in seeking medical care. This is why the disease can progress, and develop otherwise preventable complications, despite having low mortality if treated early on. *Brucella abortus* and *Brucella melitensis* are the most frequently isolated species [4]. The diagnosis is supported by a positive echocardiogram showing valvular vegetations with positive blood cultures or identification of Brucella antibody via ELISA testing [4]. In our case, antibody testing was successful in identifying the species. In developing countries like Pakistan and India, culturing for fastidious organisms is required to isolate Brucella. This process can be challenging as it requires specific medium for culture. It has been identified based on Gram-negative coccobacilli, urease and oxidase positivity [5]. Results from blood culturing the organism have shown a sensitivity of 91% and so there is a chance for false negative results; in these cases, where there is a high suspicion, ELISA antibody tests can be utilized. The incidence of developing IE is rare ranging up to 4% but it is the most notorious complication of brucellosis Infection. [4]. When it does occur, however, mortality from the disease can rise significantly to 80% [6]. There is no specific treatment geared towards Brucella infective endocarditis since the incidence is so rare. The management varies and the current recommendation includes rifampin 600 mg daily and doxycycline 100mg twice daily for a minimum of six weeks [7]. In addition, IV formulations of gentamicin or ceftriaxone may be added for a further two weeks for patients who develop infective endocarditis. Following medical management, surgery is usually required to repair the damaged valves within four weeks to three months [8].

## Conclusions

Brucellosis is prevalent in developing countries but a relatively benign disease if treated appropriately. Unfortunately, due to vague symptoms and non-specific laboratory values, it can be frequently missed and progress leading to fatal complications. Infective endocarditis is one such complication which can greatly increase the mortality rate up to 80%. In such cases, treatment with IV and oral antibiotics can be indicated along with surgical intervention for any findings of cardiac vegetations on imaging. Appropriate education regarding proper hygiene and food handling, especially in more rural settings containing farms, livestock, and access to unpasteurized milk, may help raise awareness and hopefully expedite treatment initiation. 

## References

[1] Pappas G, Papadimitriou P, Akritidis N, Christou L, Tsianos EV (2006). The new global map of human brucellosis. Lancet Infect Dis.

[2] Ducrotoy MJ, Bertu WJ, Ocholi RA, Gusi AM, Bryssinckx W, Welburn S, Moriyón I (2014). Brucellosis as an emerging threat in developing economies: lessons from Nigeria. PLoS Negl Trop Dis.

[3] Kang GJ, Gunaseelan L, Abbas KM (2014). Epidemiological modeling of bovine brucellosis in India. Proc IEEE Int Conf Big Data.

[4] Raju IT, Solanki R, Patnaik AN, Barik RC, Kumari NR, Gulati AS (2013). Brucella endocarditis - a series of five case reports. Indian Heart J.

[5] Xu N, Wang W, Chen F, Li W, Wang G (2020). ELISA is superior to bacterial culture and agglutination test in the diagnosis of brucellosis in an endemic area in China. BMC Infect Dis.

[6] Hadjinikolaou L, Triposkiadis F, Zairis M, Chlapoutakis E, Spyrou P (2001). Successful management of Brucella mellitensis endocarditis with combined medical and surgical approach. Eur J Cardiothorac Surg.

[7] (1986). Joint FAO/WHO expert committee on brucellosis. World Health Organ Tech Rep Ser.

[8] Jacobs F, Abramowicz D, Vereerstraeten P, Le Clerc JL, Zech F, Thys JP (1990). Brucella endocarditis: the role of combined medical and surgical treatment. Rev Infect Dis.

